# CO_2_ Hydrogenation over Unsupported Fe-Co Nanoalloy Catalysts

**DOI:** 10.3390/nano10071360

**Published:** 2020-07-11

**Authors:** Marco Calizzi, Robin Mutschler, Nicola Patelli, Andrea Migliori, Kun Zhao, Luca Pasquini, Andreas Züttel

**Affiliations:** 1 Laboratory of Materials for Renewable Energy, Institute of Chemical Sciences and Engineering, École Polytechnique Fédérale de Lausanne, 1951 Sion, Switzerland; marco.calizzi@gmail.com (M.C.); robin.mutschler@empa.ch (R.M.); kun.zhao@epfl.ch (K.Z.); andreas.zuettel@epfl.ch (A.Z.); 2 EMPA Materials Science & Technology, 8600 Dübendorf, Switzerland; 3 Department of Physics and Astronomy, Alma Mater Studiorum Università di Bologna, 40127 Bologna, Italy; luca.pasquini@unibo.it; 4 Unit of Bologna, Institute of Microelectronics and Microsystems, National Research Council, 40129 Bologna, Italy; migliori@bo.imm.cnr.it

**Keywords:** nanoparticle, nanoalloy, catalyst, CO_2_ reduction, hydrocarbon, synthetic fuel, iron, cobalt

## Abstract

The thermo-catalytic synthesis of hydrocarbons from CO_2_ and H_2_ is of great interest for the conversion of CO_2_ into valuable chemicals and fuels. In this work, we aim to contribute to the fundamental understanding of the effect of alloying on the reaction yield and selectivity to a specific product. For this purpose, Fe-Co alloy nanoparticles (nanoalloys) with 30, 50 and 76 wt% Co content are synthesized via the Inert Gas Condensation method. The nanoalloys show a uniform composition and a size distribution between 10 and 25 nm, determined by means of X-ray diffraction and electron microscopy. The catalytic activity for CO_2_ hydrogenation is investigated in a plug flow reactor coupled with a mass spectrometer, carrying out the reaction as a function of temperature (393–823 K) at ambient pressure. The Fe-Co nanoalloys prove to be more active and more selective to CO than elemental Fe and Co nanoparticles prepared by the same method. Furthermore, the Fe-Co nanoalloys catalyze the formation of C_2_-C_5_ hydrocarbon products, while Co and Fe nanoparticles yield only CH_4_ and CO, respectively. We explain this synergistic effect by the simultaneous variation in CO_2_ binding energy and decomposition barrier as the Fe/Co ratio in the nanoalloy changes. With increasing Fe content, increased activation temperatures for the formation of CH_4_ (from 440 K to 560 K) and C_2_-C_5_ hydrocarbons (from 460 K to 560 K) are observed.

## 1. Introduction

CO_2_ capture and utilization (CCU) is the process of capturing CO_2_ anthropogenic emissions and using them to synthesize valuable and useful chemicals. When applied to fuel production, this concept translates into a closed carbon cycle, thus implementing a sustainable energy system. The production of liquid synthetic fuels is especially interesting for large scale energy storage because they retain all the benefits of liquid fossil fuels, such as high energy density and stability in ambient conditions [1,2]. In this framework, the quest for a material that efficiently catalyzes the reaction between CO_2_ and H_2_ is of key importance, since CO_2_ is a very stable molecule ($\Delta_{f}H_{298\;K}^{0}{,}_{{\mathrm{CO}}_{2}}=-393.5\;\mathrm{kJ}/\mathrm{mol})$.

Historically, there are two major reactions for the thermo-catalytic synthesis of hydrocarbons from CO_2_ or CO: The Sabatier reaction (Equation (1)), which is highly selective towards CH_4_, and the Fischer–Tropsch (FT) reaction (Equation (2)), which is up to date and the most industrially relevant reaction to synthesize hydrocarbon fuels, alcohols and waxes from syngas (mixture of H_2_ and CO, mainly). If CO_2_ obtained from the atmosphere or from local emitters is the starting molecule for the synthesis, the FT reaction can be combined with the (endothermic) reverse water gas-shift reaction (RWGS, Equation (3)). Another variation is the direct conversion of CO_2_ to higher hydrocarbons via a FT-like reaction (Equation (4)).

(1) $${\mathrm{CO}}_{2}+4H_{2}\to {\mathrm{CH}}_{4}+2H_{2}O\;(\Delta H^{0}=-164.9\;\mathrm{kJ}/\mathrm{mol}),$$

(2) $$\mathrm{nCO}\;+\left(2n\;+1\right)H_{2}\to C_{n}H_{2n+2}+{\;\mathrm{nH}}_{2}O,$$

(3) $${\mathrm{CO}}_{2}+{\;H}_{2}\to \mathrm{CO}\;+{\;H}_{2}O\;(\Delta H^{0}=+41.18\;\mathrm{kJ}/\mathrm{mol}),$$

(4) $${\mathrm{nCO}}_{2}+(3n\;+1)H_{2}\to C_{n}H_{2n+2}+2{\mathrm{nH}}_{2}O,$$

The C_2+_ products are energetically close together, therefore, the catalyzed synthesis leads to a wide range of products. Furthermore, the synthesis of C_2+_ products requires the reaction of CO_2_ with hydrogen and simultaneously also the reaction between the C-containing intermediates. These two competing reactions have to be controlled independently in order to yield a specific product. The direct CO_2_ hydrogenation is of great importance and recent publications have shown that the selectivity and yield towards C_2+_ hydrocarbons can be increased either via the combination of different catalysts in a multi-catalysts bed [3,4,5], or via alloying the transition metals Fe, Co, Ni and Cu, combined with alkali metal promoters, such as Na and K [6]. 

Fe-based alloys, such as Fe-M (M = Cu, Co, Ni), have been in the focus of investigations since they were found to be the most active elements of industrial relevance in the formation of longer chained HCs via direct CO_2_ hydrogenation [7,8,9]. Among these kinds of materials, supported Fe-Co-based nanoparticles (NPs) show the highest C_2+_ yields: 25.4% C_2+_ yield with 35.8% CO_2_ conversion for K-promoted Fe_0.9_Co_0.1_ on Al_2_O_3_ [10], or 14.3% C_2+_ yield with 33.3% CO_2_ conversion for Fe_0.9_Co_0.1_ on TiO_2_ (1.1 MPa, 573 K) [9]. 

While the supporting metal oxide phases are known to alter the reaction, they also provide mechanical and thermal stability to the NPs to avoid sintering [11]. The mainly alkali metal promoters are used to enhance the selectivity towards C_2+_ hydrocarbons. 

The principal focus of the literature is on finding the material with the best catalytic activity and selectivity in order to increase the reaction yield. In this work, instead, we aim to contribute to the fundamental understanding of the effect of alloying on the reaction yield and selectivity to a specific product. Therefore, to study the fundamental influence of alloying Fe and Co, we relinquish the use of a metal oxide support and promoters. The aim of this paper is the analysis of the structure, composition and stability of unsupported Fe-Co alloy NPs (nanoalloys), synthesized via Inert Gas Condensation (IGC), and their catalytic properties in the CO_2_ hydrogenation reaction. 

IGC is chosen as synthesis method since it is a versatile technique that can produce elemental NPs [12], bimetallic NPs [13,14], and other hydride or oxide nanocomposites [15,16] of high purity, with good control of the bulk composition in free-standing powder form.

## 2. Materials and Methods 

NPs were grown via IGC in an ultrahigh-vacuum (UHV) chamber, equipped with a tungsten boat as a thermal vapor source [17]. The precursor material of the Fe-Co nanoalloys was a mixture of microcrystalline Fe (Sigma-Aldrich, Darmstadt, Germany, particle size <450 μm, purity >99%) and Co (Sigma-Aldrich, particle size <150 μm, purity ≥99.9%) powders previously melted and alloyed in the tungsten boat. Three Fe-Co powder mixtures with 25 wt%, 50 wt% and 75 wt% Co content were used to synthesize nanoalloys with different Fe/Co ratios. The corresponding samples are named 30Fe70Co, 50Fe50Co, and 76Fe24Co throughout the manuscript, according to the SEM-EDX quantified elemental wt% content of the sample, as reported in Table 1. Single-element Fe and Co NPs were also synthesized from the unmixed powders for comparison.

The synthesis chamber was preliminarily evacuated to 2 × 10^−5^ Pa and then filled with He (99.9999% purity) up to a final pressure of 260 Pa. NPs nucleated in the gas phase because the metal vapors quickly supersaturated via thermalization with the surrounding He gas. A He flow of 60 mL_n_/min, regulated with the aid of a mass flow controller, was directed on the evaporation boat during the whole synthesis in order to facilitate the removal of the NPs from the hot zone where NP coalescence takes place. The pressure was maintained constant by the simultaneous operation of the rotary pump. A liquid N_2_ cooled rotating stainless-steel cylinders allowed for the collection of NPs via thermophoresis. The NPs were finally scraped off by means of a stainless-steel blade and transferred into an auxiliary UHV chamber, from which they were extracted and sealed under inert Ar atmosphere.

The composition and structure of the as-prepared NPs were determined by scanning electron microscopy (SEM) with a Leica Cambridge Stereoscan 360 equipped with an X-ray detector for energy dispersive X-ray (EDX) microanalysis, and by X-ray diffraction (XRD) with a PANalytical X’celerator diffractometer employing Cu Kα radiation. XRD patterns were analyzed with the MAUD Rietveld refinement software [18] to determine the lattice parameters, crystallite size, and relative phase abundance.

The morphology and the size distribution of the samples were analyzed by means of a FEI Tecnai F20 ST transmission electron microscope (TEM), operated at 200 kV. High-angle annular dark field (HAADF) images and EDX elemental profiles at the single NP level were acquired in the scanning TEM mode (STEM). For TEM analysis, the NPs were dispersed in isopropanol and the suspension was drop-casted on a holey carbon support grid. The specific surface areas of the nanoalloys were determined by applying the Brunauer–Emmett–Teller (BET) method to N_2_ adsorption isotherms measured in a Belsorp mini II instrument after degassing in a vacuum at 423 K for 0.5 h.

The CO_2_ hydrogenation experiments were carried out in a dedicated gas control and analysis system with a highly isothermal packed bed [19]. The reactor was loaded with 10 mg of catalyst in the glovebox and the reactor tubing was pumped out and flushed with He three times before the valves that connect the reactor to the tubing were opened. After opening the valves, a constant He flow of 10 mL_n_/min was applied and the reactor was heated up to 393 K. 

The catalyst and the reactor tubing were pre-treated in these conditions for at least 30 min to evaporate remaining moisture. Furthermore, all the tubes in the downstream were heated to 433–473 K in order to evaporate the moisture in the tubing to the mass spectrometer. After the He pretreatment, the catalyst and tubing were treated with a 7.5 mL_n_/min H_2_ and 2.5 mL_n_/min He gas mixture at 393 K to further reduce remaining oxides. The nanoalloys were not pre-reduced at high temperature to avoid coarsening phenomena that would lead to the loss of the nanostructure. They were dispersed on glass-wool to facilitate the loading into the reactor and to distribute evenly in the reactor tube.

The aluminum inlet in the reactor oven ensured a uniform temperature distribution over the length of the reactor and, therefore, enabled a precise temperature measurement by means of a thermocouple placed directly on the reactor tube. 

After the surface reduction, a gas mixture with a ratio of H_2_:CO_2_ = 4:1 with He as carrier gas and a total flow of 10 mL_n_/min was set on the mass flow controllers to the bypass of the reactor after closing the valves to the reactor. The gas flows and purities were: 6 mL_n_/min and 99.995% for H_2_; 1.5 mL_n_/min and 99.998% for CO_2_; 2.5 mL_n_/min and 99.999% for He. After a stable gas mixture was achieved, the reaction gas stream was let through the reactor and the measurement was started. The reactor oven was then heated up at a rate of 2 K/min from 393 K to 823 K (oven set points). The effective measured temperatures were 390 K to 810 K on the reactor. The reaction was carried out at ambient pressure. Given the small amount of sample, eventual gaseous products arising from further reductions in phases in the material during the temperature ramp would be negligible, compared to the gas flow in the reactor.

A spectrum of masses from 1 to 100 u was measured approximatively every two minutes with an OmniStar Pfeiffer Mass Spectrometer (MS). The product analysis by means of MS is discussed in the next section.

For the analysis of the products of the catalytic reaction, we applied a semi-quantitative method by means of mass spectrometry (MS), which allowed for the comparison of the activity and selectivity among the catalysts. This method is a variation of the quantitative analysis method we developed and described in detail in our previous work [19,20]. In comparison to that method, we accounted for the pressure fluctuations over the MS capillary by normalizing the intensity of the signal to a reference pressure, which was defined and the same for all experiments. The internal pressure was logged for every spectrum; hence the normalization was applied on each spectrum. Since the signal intensity in the Faraday detector was linear to the incident ions, it was also directly proportional to the pressure difference over the capillary. Therefore, a normalized and comparable signal was obtained if the signal intensity (ion current) was multiplied by a correction factor $f_{corr},$ calculated from the logged MS partial pressure $p_{\mathrm{MS}}$ and the reference pressure $p_{ref}$:

(5) $$f_{corr}=p_{MS}/p_{ref}$$

where $p_{ref}=10^{-3}\;\mathrm{Pa}$. After the integration of the peaks over one half of a mass/charge ratio *m*/*z*, the integration area was normalized with $f_{corr}$:

(6) $$A_{corr,mz}=A_{mz}/f_{corr}$$

where $A_{\mathrm{mz}}$ is the area of the integrated signal peak for one *m*/*z*. With this method, the (integrated) signal intensity for different catalysts could be directly compared for each *m*/*z*, allowing a semi-quantitative analysis. For the quantification of the partial pressures of the products with this method, a pressure-normalized calibration for all products would be required. For our purposes, this was not necessary.

In any case, the MS peaks of C1-C5 hydrocarbons (HCs) strongly overlapped with the peaks of CO_2_ and CO. To identify the ideal *m*/*z* peaks for the analysis, reference electron ionization patterns for each compound (C1-C5 HC, MeOH, EtOH, CO, CO_2_, H_2_ and He) were plotted, based on the data reported on the NIST Chemistry WebBook [21]. Based on these data, the reference peaks were selected, as shown in Table S1.

## 3. Results

### 3.1. Structure, Morphology and Composition of Fe-Co Nanoalloys

**Figure 1:**
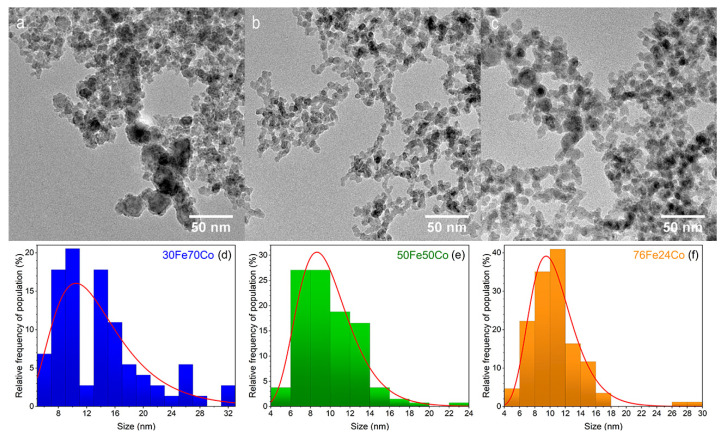
TEM images of xFe(100-x) Co samples with x = 30, 50, 76 in frames (**a**–**c**), respectively. The corresponding NPs size distributions are given in frames (**d**–**f**).

The TEM images in Figure 1a–c show the morphology of the as-prepared nanoalloys. The NPs sizes follow a log-normal distribution, typical for this technique [22]. The mean NP size spans from 10 to 13 nm, with sample 30Fe70Co having the largest size. It is worth noting that the distributions in Figure 1d,f are, intrinsically, number-weighted distributions. However, in Table 1 we report the volume-weighted mean diameter (or de Brouckere mean ${\bar{d}}^{TEM}$), assuming spherical particles that can be directly compared to the mean crystallite determined by XRD.

**Figure 2:**
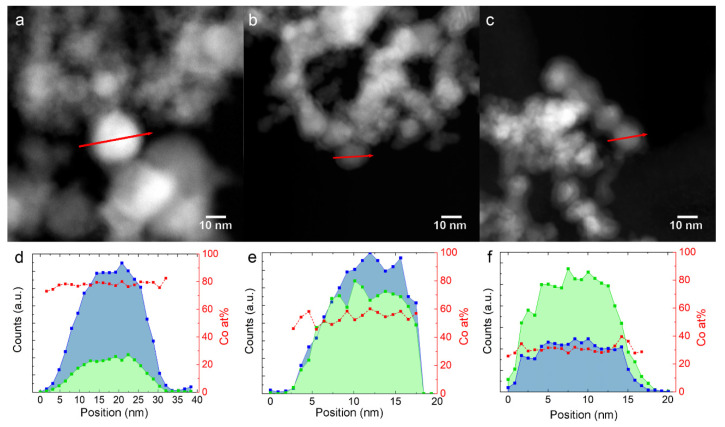
Fe and Co EDX composition profiles along the red arrow, shown in the STEM image above them. (**a**,**d**): 30Fe70Co; (**b**,**e**): 50Fe50Co; (**c**,**f**): 76Fe24Co. The blue and green profiles represent Co and Fe counts, respectively. Red squares represent the calculated Co atomic content.

The EDX profiles in Figure 2d,f have been measured on Fe-Co nanoalloys along the red path, indicated in the HAADF-STEM images in Figure 2a–c, respectively. The red squares in the graphs show that the Co/(Co + Fe) ratio is homogeneous from the core to the surface within the single NPs for all samples. 

The overall Fe and Co contents measured by SEM-EDX on the NPs batch are reported in Table 1. In the two samples with higher Fe content, the SEM-EDX agree with the nominal precursor composition within the experimental uncertainty. The sample 30Fe70Co seems to have a higher Fe content (30 ± 2 wt%) than its precursor. This discrepancy can be explained by the higher vapor pressure of Fe (37 Pa at 2000 K) with respect to Co (20 Pa at 2000 K) [23], which leads to a higher evaporation rate of Fe. It is, however, possible to calibrate the relative Fe content in the precursor in order to achieve the target nanoalloy composition. Such an approach requires that the activity coefficients of the two elements do not vary strongly with composition, otherwise the relative evaporation rates would change during the synthesis resulting in a non-homogeneous batch. Another option is to use two closely spaced and independently controlled evaporation sources to produce a vapor mixture with the target composition [24].

The specific surface area of each sample measured with the BET method, $S_{A}^{BET}$, is reported in Table 1. Assuming spherical and isolated NPs (no interfaces), it is possible to evaluate the specific surface area $S_{A}^{TEM}\;$from the TEM size distribution through the formula:

(7) $$S_{A}^{TEM}\;=\frac{S_{TOT}}{\rho \;V_{TOT}}=\frac{6\;\sum_{i}d_{i}^{2}}{\rho \;\sum_{i}d_{i}^{3}},$$

where $d_{i}$ is the diameter of the *i*-th particle observed by TEM and ρ is the density of the NPs. The results are compared to $S_{A}^{BET}$ in Table 1. For the calculation, ρ is taken as a weighted average of the bulk densities of Fe, $\rho_{Fe}=$ 7960 kg m^−3^, and Co, $\rho_{Co}=$ 8830 kg m^−3^.

**Figure 3:**
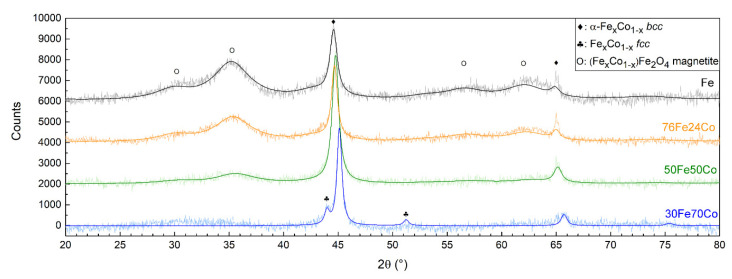
XRD patterns of the three Fe-Co nanoalloys compared to elemental Fe NPs. The peaks of the bcc Fe-Co alloy shift toward higher angles with increasing Co content.

Figure 3 shows the XRD patterns of Fe and the three nanoalloys, all showing the Bragg reflections characteristic of a body-centered cubic (bcc) α-phase. The (110) peak shifts toward higher angles with increasing Co content, indicating the decrease in the lattice parameter, as shown in Table 1, compatible with the smaller atomic radius of Co. In sample 30Fe70Co only, traces of a second face-centered cubic (fcc) Fe-Co phase are detected. A Fe_3_O_4_ magnetite-like inverse spinel structure is also detected, featuring broader diffraction peaks corresponding to a crystallite size of about 3 nm. The relative abundance of this phase within the sample decreases with increasing Co content and is barely visible in the XRD pattern of 30Fe70Co.

### 3.2. Catalytic Properties of Fe-Co Nanoalloys

**Figure 4:**
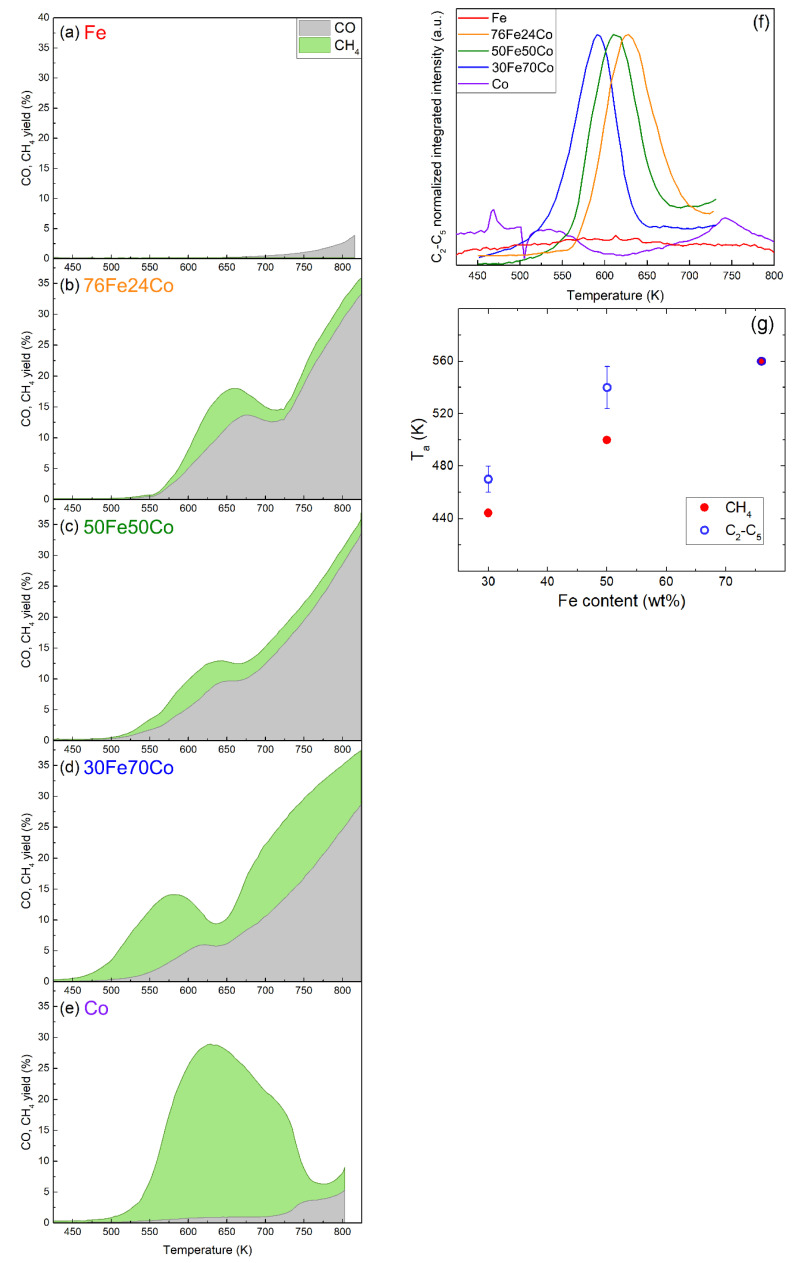
Catalytic properties of samples (**a**) Fe, (**b**) 76Fe24Co, (**c**) 50Fe50Co, (**d**) 30Fe70Co, (**e**) Co in a flow reactor with 4:1 H_2_:CO_2_ ratio, 1 bar and 10 mL_n_ min^−1^, measured by mass spectroscopy. The stacked area-filled plots show the total CO_2_ conversion with the separated contributions of CO yield in grey and CH_4_ yield in green; (**f**) conversion of CO_2_ into C_2_-C_5_ HCs obtained semi-quantitatively by summing the normalized MS signals for *m*/*z* = 26, 29, 30, 39, 56, 57, and 70; (**g**) activation temperature $T_{a}$ of the nanoalloy catalysts for CH_4_ and C_2_-C_5_ production as function of their Fe content. The error bars represent the standard deviation in the activation temperatures of the different C_2_-C_5_ HCs, as detailed in Table S2.

The catalytic performances of the samples are summarized in Figure 4a–e, showing the CO_2_ conversion, CO yield and CH_4_ yield for the nanoalloys and elemental NPs. Here the yield is defined as the ratio between the output flow of the product and the input flow of CO_2_, meaning that the sum of all product yields is equal to the CO_2_ conversion. A product-by-product comparison of the same results is presented in Figure S1 of the SI. Figure 4f shows the C_2_-C_5_ yield of the same samples. The Fe-Co nanoalloys all exhibit activity in the CO_2_ hydrogenation reaction where CH_4_ and CO are the major carbon containing products on all catalysts. Compared to elemental Fe or Co NPs, the nanoalloys show conversion yields for CO and CH_4_ with a different temperature dependence and genuinely new activity towards the formation of C_2_-C_5_ hydrocarbons. The total CO_2_ conversion is given as the sum of CO and CH_4_ yields. As explained in the Materials and Methods section, the analysis of C_2_-C_5_ hydrocarbons is semi-quantitative but the absolute yield would be < 1%, therefore neglecting it does not significantly affect the CO_2_ conversion curve. The formation of alcohols, such as methanol or ethanol, is not detected. The highest CO_2_ conversion (at moderate temperatures) is 18%, achieved by 76Fe24Co at 662 K with 28% CH_4_ and 72% CO selectivity. While the effects of the alloy composition on the reaction temperatures will be discussed later, no clear trend for the maximum conversion of C_2_-C_5_ products as a function of the Fe content in the alloy can be determined.

Figure 4g reports the activation temperatures $T_{a}$ for the formation of CH_4_ and C_2_-C_5_ HCs products, as determined by analyzing the reference MS peaks for each molecule versus the Fe content in the nanoalloys. The error bar given on the data points for C_2_-C_5_ corresponds to the standard deviation of the $T_{a}$ values observed for the various products, as shown in Table S2.

Two trends are observed: first, the formation of C_2_-C_5_ HCs starts at temperatures higher than those observed for CH_4_, with the exception of 76Fe24Co, where $T_{a}=560$ K for all HCs. Second, $T_{a}$ increases with increasing Fe content in the alloy. This is also clearly visible in Figure 4a–e for CH_4_ and in Figure 4f for C_2_-C_5_ products, where the peaks shift to higher temperatures with increasing Fe content. Table S2 in the SI lists the detailed results of the kinetic analysis for the three nanoalloys, reporting the kinetic reaction range, the temperature of maximum activity, and the activation energy $E_{a}$ of each product.

As concerns $E_{a}$, in almost all cases, it is larger for the C_2_-C_5_ products than for CH_4_, as listed in Table S2. Moreover, Figure S2 shows that the $E_{a}$ values of the C_2_-C_5_ products increase with increasing Fe content, thus following a trend similar to $T_{a}$. The $E_{a}$ values are in the ranges 65–80 kJ mol^−1^ for Fe30Co70, 80–140 kJ mol^−1^ for Fe50Co50, and 110–275 kJ mol^−1^ for Fe76Co24. It is worth noting that these values are measured at conditions constrained by the thermal stability of the materials and are meant to be compared within this work, not to other Fe-based catalysts that have been properly activated with thermal treatments that typically last several hours above 573 K.

## 4. Discussion

### 4.1. Structure, Morphology, and Composition of Fe-Co Nanoalloys

The volume-weighted average NPs size ${\bar{d}}^{TEM}$ calculated from the size distributions of Figure 1 under the assumption of spherical NPs is in good agreement with the average crystallite size, determined by XRD, as shown in Table 1, supporting the idea that NPs are mostly single crystalline. The specific surface area $S_{A}^{TEM}$, estimated from the TEM size distributions under the additional hypothesis of isolated NPs, is in good agreement with the BET result $S_{A}^{BET}$ for the two samples, 76Fe24Co and 50Fe50Co, suggesting that NPs retain most of their free surface despite aggregation. Contrarily, for sample 30Fe70Co, $S_{A}^{TEM}$ is about 50% larger than $S_{A}^{BET}$, indicating that aggregation leads to a loss of surface area in favor of interface area. This is in qualitative agreement with the presence of NPs in Figure 1a, the size of which largely exceeds the average value, hinting at inter-particle coalescence. 

HAADF-STEM images, as shown in Figure 2a–c, show that the NPs are surrounded by a 2–3 nm thick shell with a lower contrast, which suggests that the average atomic number is lower than in the core. This information, combined with XRD, points to the metal core/oxide shell nature of the NPs. The oxide shell has a cubic inverse spinel structure (space group $Fd\bar{3}m$) and forms during the slow air exposure for XRD and TEM experiments [25]. The shell is identified as magnetite Fe_3_O_4_ for the elemental Fe NPs, and cobalt ferrite (Fe_x_Co_1-x_)_3_O_4_ [26,27] for the Fe-Co nanoalloys. The STEM-EDX profiles in Figure 2e,f show that the Fe/Co ratio in the shell is similar to the core. The (Fe_x_Co_1-x_)_3_O_4_ peaks decrease in intensity with increasing Co content, indicating a higher oxidation resistance, as already reported for nearly equimolar Fe-Co nanoalloys [28].

After the CO_2_ hydrogenation experiments the oxide, is not detected anymore by XRD, as shown in Figure S3, nor is carbon laydown. The mean crystallite size is increased as expected after exposure to temperatures >800 K; however, the alloy is still observed with no phase segregation.

### 4.2. Catalytic Properties of Fe-Co Nanoalloys: Compositional Effects

The Fe-Co alloys, compared to elemental Fe and Co, display a much higher selectivity toward CO, an intermediate one for CH_4_, and show activity in the catalytic formation of C_2_-C_5_ hydrocarbons, as shown in Figure 4f. The high activity of the Fe-Co alloys towards CO means that the surface of these catalysts is rich in adsorbed CO, which is not the case for Fe and Co, although for opposite reasons, as discussed later. The abundance of adsorbed CO suggests that the C_2_-C_5_ HCs are synthesized via RWGS + FT reactions (Equations (2) and (3)) rather than via direct CO_2_ hydrogenation (Equation (4)). Remember that Co acts as a purely Sabatier catalyst, while Fe is active towards the RWGS reaction, and only at high temperatures.

**Figure 5:**
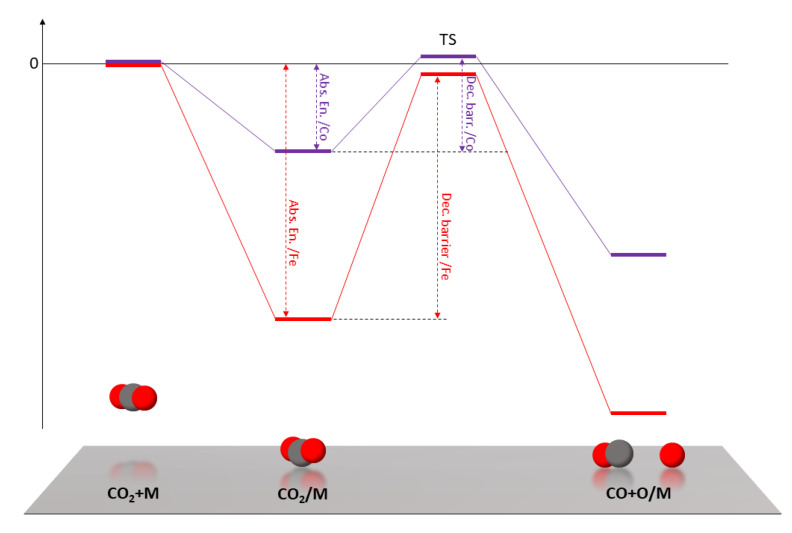
Schematic representation of the calculated energy levels of (from left to right) free CO_2_, adsorbed CO_2_, transition state, decomposed CO_2_ into CO + O. In red, relative to Fe surface; in purple, relative to Co surface. The dashed vertical lines highlight the difference between Fe and Co in adsorption energy and decomposition barrier [29].

The theoretical work of Liu et al. [29] on the reduction of CO_2_ on transition metal surfaces provides a deeper understanding of these results. They conclude that CO_2_ is strongly adsorbed on Fe surfaces, but this is not favorable for CO_2_ decomposition into CO + O, because the energy of the transition state between adsorption and decomposition causes a high reaction barrier. On the other side, CO_2_ is weakly adsorbed on Co surfaces, but the transition state energy is similar to that of Fe, resulting in a lower decomposition barrier. A qualitative representation of the relevant energy levels and barriers is sketched in Figure 5. The adsorption and decomposition of the CO_2_ molecule are necessarily the first steps in CO_2_ hydrogenation and here we argue that they are key steps for the Fe-Co catalytic system. Based on the results of these calculations, it is possible to better interpret the catalytic activity measured for pure Fe and Co. Fe surfaces are covered with CO_2_ that is easily adsorbed but is too strongly bound to react, showing very poor CO_2_ conversion rates in general. When the temperature is high enough to overcome the CO_2_ decomposition barrier, CO is the only product because it is thermodynamically favored. On Co surfaces, the weak adsorption energy means that the surface is not rich in adsorbed CO_2_ but most of the adsorbed molecules decompose into CO because of the low decomposition energy barrier. The few and isolated CO molecules cannot then meet to start the HC chain by forming C-C bonds, so that the hydrogenation reaction necessarily goes on from CO to CH_4_.

Although we are not aware of similar theoretical studies for Fe-Co surfaces, it is reasonable to expect a composition-dependent intermediate behavior for the alloy. This view is supported by our experimental finding that both $E_{a}$ and $T_{a}$ of HCs formation increase with increasing Fe content in the alloy. Higher $E_{a}$ and $T_{a}$ values mean a stronger interaction of the adsorbed species with the surface (i.e., a more Fe-like behavior). By adjusting the Fe/Co ratio in the alloy, it is therefore possible to tune the CO_2_ adsorption energy and decomposition barrier in order to have more CO_2_ adsorbed than on Co but, at the same time, an easier decomposition to CO than on Fe. The result is an abundance of adsorbed CO on the alloy surface, which is the cause for the higher selectivity to CO and the starting point in the formation of C-C bonds for the growth of HC chains in FT synthesis. The low C_2_-C_5_ yields (in Figure 4f the *y*-axis is in arbitrary units, but the yield is < 1%), the limitations of the MS technique, and the limited amount of data do not allow for a quantitative composition dependent analysis. It will be interesting to investigate this aspect in a future work and at higher operating pressures.

The $T_{a}$ values of the various C_2_-C_5_ products are similar and slightly higher than for CH_4_ ($\Delta T_{a}\sim$35 K for 30Fe70Co and 50Fe50Co). This difference in $T_{a}$ is explained considering the superior kinetics of the Sabatier reaction compared to the RWGS + FT reaction [30], especially at lower temperatures, where the Sabatier reaction is also thermodynamically favored. With increasing temperature, CO synthesis through RWGS starts to compete with the Sabatier reaction. This is the reason why $\Delta T_{a}$ almost vanishes for the 76Fe24Co nanoalloy (i.e., the composition that shows the highest activation temperature $T_{a}=560\;K$).

## 5. Conclusions

The Fe-Co nanoalloys synthesized by inert gas condensation exhibit enhanced catalytic activities for CO_2_ hydrogenation compared to elemental Fe and Co NPs, being also slightly active toward the synthesis of C_2_-C_5_ hydrocarbons. On top of CO_2_ conversion and product yield, thanks to the developed set-up, based on mass spectrometry, it is possible to measure the activation temperature $T_{a}$ and estimate the activation energy $E_{a}$ for each reaction product. The observed increase in both $T_{a}$ and $E_{a}$ with rising Fe content in the nanoalloys, as well as their activity in C_2_-C_5_ synthesis, is interpreted based on the idea of composition-dependent CO_2_ adsorption energy and decomposition barrier. The balance between the high density of adsorbed stable CO_2_, typical of Fe, and the low density of easily decomposed CO_2_, typical of Co, leads to higher activity and C-C bond formation on the Fe-Co surface, which initiates the hydrocarbon chain. This work is relevant in assessing the catalytic properties of Fe-Co nanoalloys, ruling out the effects of supports, metal/support interfaces, and promoters.

## Figures and Tables

**Table 1:** Mean crystallite size $\bar{d}$ and lattice parameter a obtained from XRD analysis of metallic bcc reflections; Fe and Co content from EDX; BET-measured surface area $S_{A}^{BET}$; surface area $S_{A}^{TEM}$ and volume-weighted mean diameter ${\bar{d}}^{TEM}$ calculated from TEM size distributions. The numbers in parenthesis represent the standard error in units of the last significant digit.

Sample	$\bar{d}$	a (Å)	wt% Fe	wt% Co	$S_{A}^{BET}\;(m^{2}\;g{-}^{1})$	$S_{A}^{TEM}\;(m^{2}\;g{-}^{1})$	${\bar{d}}^{TEM}\;(\mathrm{nm})$
**Fe**	15 (1)	2.8729 (4)	100	0	--	--	--
**76Fe24Co**	19 (1)	2.8686 (2)	76 (2)	24 (2)	47 (9)	56	15 (7)
**50Fe50Co**	18 (1)	2.8629 (2)	50 (2)	50 (2)	56 (5)	63	13 (5)
**30Fe70Co**	25 (1)	2.8410 (2)	30 (2)	70 (2)	56 (5)	37	22 (7)

## Supplementary Materials

The following are available online at https://www.mdpi.com/2079-4991/10/7/1360/s1. Table S1: Reference table for the assignments of mass spectrometer peaks. Table S2. Activation temperature ($T_{a}$) of selected m/z mass spectrometer peaks, which are assigned to C_1_-C_5_ hydrocarbon products, the corresponding temperature of the maximum activity Tmax, the starting and ending temperature of the kinetically determined reaction range (T1 kin and T2 kin), and activation energy ($E_{a}$). %Tmax is an indicator that tells us if the reaction is solely limited by the reaction kinetics; it gives the ratio of T2 kin compared to Tmax. R^2^ is assigned to the Arrhenius plots (inverse T1 kin to T2 kin vs. the natural logarithm of the normalized MS signal) on which the activation energy is determined. Figure S1: Catalytic properties of Fe, Co and Fe-Co NPs in a flow reactor with 4:1 H_2_:CO_2_ ratio, 1 bar and 10 mL_n_ min^−1^, measured by mass spectroscopy. (**a**) CO_2_ conversion (**b**) CO yield, (**c**) CH_4_ yield, (**d**) conversion curves of the summed up C2-C5 mass spectrometer normalized signals for m/z = 26, 29, 30, 39, 56, 57, 70. Figure S2: Activation energies of C2-C5 product formation as a function of the Fe content in the alloy precursor. The colors corresponding to the different products are listed in the legend, with the m/z ratio of the MS reference peak increasing from top to bottom. Figure S3 (a): XRD of the Fe-Co samples after the CO_2_ hydrogenation experiments, background corrected; (b): a detail of the main Fe-Co peak shifted to higher angular positions with increasing Co content.
