# Supplementary material for: CO2 Hydrogenation over Unsupported Fe-Co Nanoalloy Catalysts
Source: Nanomaterials (Basel). 2020 Jul 11;10(7):1360. doi: 10.3390/nano10071360 (PMC7408123; doi:10.3390/nano10071360)
Supplement: Supplementary file 1 [file nanomaterials-10-01360-s001.pdf]

Supplementary Information

CO_2 hydrogenation over unsupported Fe-Co nanoalloy catalysts

Marco Calizzi [1,2], Robin Mutschler [1,2], Nicola Patelli [3,*], Andrea Migliori [4], Kun Zhao [1,2], Luca Pasquini [3], and Andreas Züttel [1,2]

[1] Laboratory of Materials for Renewable Energy, Institute of Chemical Sciences and Engineering, École polytechnique fédérale de Lausanne, Valais/Wallis, Energypolis, Sion, Switzerland
[2] EMPA Materials Science & Technology, Dübendorf, Switzerland
[3] Department of Physics and Astronomy, Alma Mater Studiorum Università di Bologna, Bologna, Italy
[4] Unit of Bologna, Institute of Microelectronics and Microsystems, National Research Council, Bologna, Italy
* Correspondence: nicola.patelli@unibo.it

Mass spectrometry

Table S1. Reference table for the assignments of mass spectrometer peaks.

**Molecule**	**Major peak of molecule [m/z]** (100% relative intensity)	**Reference peak for molecule[m/z]**	**Relative intensity reference peak [%]**	**Minor contributions to peak. Relative intensity to the molecules major peak (greater than 5%)**
CH_4	16	15	89	MeOH (12%), EtOH (7%)
C_2H_4	28	26	53	C_2H_6 (23%), C_3H_6 (10%), C_3H_8 (9%), C_4H_8 (8%), EtOH (10%)
C_2H_6	28	30	26	MeOH (6%), EtOH (8%)
C_3H_6	41	39	73	C_3H_8 (18%), C_4H_8 (34%), C_4H_{10} (17%), C_5H_{10} (14%), C_5H_{10} (27%), C_5H_{12} (19%),
C_3H_8	29	29	100	C_2H_6 (22%), C_4H_8 (13%), C_5H_{10} (21%), C_5H_{12} (14%), MeOH (45%), EtOH (30%)
C_4H_8	41	56	39	None
C_4H_{10}	43	43	100	C_3H_8 (18%), C_5H_{12} (100%)
C_5H_{10}	42	70	39	None
C_5H_{12}	43	57	20	None
CO_2	44	44	100	C_3H_8 (27%)
CO	28	28	100	C_2H_4 (100%), C_2H_6 (100%), C_3H_8 (59%), C_4H_8 (27%), CO_2 (10%)
H_2O	18	18	100	None
MeOH	31	32	74	None
EtOH	31	45	51	None

Activation energy determination

Table S2. Activation temperature (T_a) of selected m/z mass spectrometer peaks, which are assigned to C_1-C_5 hydrocarbon products, the corresponding temperature of the maximum activity Tmax, the starting and ending temperature of the kinetically determined reaction range (T1 kin and T2 kin) and activation energy (E_a). %Tmax is an indicator that tells if the reaction is solely limited by the reaction kinetics; it gives the ratio of T2 kin compared to Tmax. R^2 is assigned to the Arrhenius plots (inverse T1 kin to T2 kin vs. the natural logarithm of the normalized MS signal) on which the activation energy is determined.

Sample 30Fe70Co.

m/z	ass. comp.	T_a [K]	Tmax [K]	T1 kin [K]	T2 kin [K]	Width kin [K]	% Tmax	Max signal	E_a [kJ/mol]	R^2
15	CH_4	444	570	444	525	81	64%	9.47E-10	70	0.999
26	C_2H_4	480	600	461	570	109	75%	7.39E-11	80	0.997
29	C_3H_8	470	590	529	578	49	90%	1.66E-10	64	0.998
30	C_2H_6	480	590	489	570	81	82%	5.26E-11	67	0.999
39	C_3H_6	480	600	546	583	37	86%	7.32E-11	101	0.998
56	C_4H_8	460	600	534	591	57	94%	1.98E-11	89	0.997
57	C_5H_{12}	460	590	534	583	49	95%	7.28E-12	41	0.987
70	C_5H_{10}	460	595	534	587	53	94%	6.14E-12	67	0.982

Sample 50Fe50Co.

m/z	ass. comp.	T_a [K]	Tmax [K]	T1 kin [K]	T2 kin [K]	Width kin [K]	% Tmax	Max signal	E_a [kJ/mol]	R^2
15	CH_4	500	615	499	581	82	70%	3.41E-10	75	0.992
26	C_2H_4	530	620	540	581	41	57%	5.04E-11	138	0.988
29	C_3H_8	520	620	565	590	25	70%	1.14E-10	83	0.995
30	C_2H_6	530	620	573	590	17	67%	2.54E-11	105	0.99
39	C_3H_6	530	615	540	581	41	60%	5.24E-11	160	0.984
56	C_4H_8	560	610	557	581	24	42%	1.49E-11	165	0.992
57	C_5H_{12}	550	585	561	573	12	66%	6.20E-12	141	0.979
70	C_5H_{10}	560	610	557	577	20	34%	5.28E-12	135	0.992

Sample 76Fe24Co.

m/z	ass. comp.	T_a [K]	Tmax [K]	T1 kin [K]	T2 kin [K]	Width kin [K]	% Tmax	Max signal	E_a [kJ/mol]	R^2
15	CH_4	560	645	566	599	33	46%	5.09E-10	137	0.998
26	C_2H_4	560	640	557	591	34	39%	5.95E-11	241	0.991
29	C_3H_8	560	630	574	603	29	61%	1.44E-10	109	0.996
30	C_2H_6	560	635	566	603	37	57%	4.36E-11	161	0.996
39	C_3H_6	560	630	557	578	21	26%	4.82E-11	277	0.996
56	C_4H_8	560	620	557	578	21	30%	1.39E-11	228	0.992
57	C_5H_{12}	560	600	557	578	21	45%	8.23E-12	198	0.99
70	C_5H_{10}	560	605	562	574	12	31%	5.03E-12	226	0.988

Catalytic properties: product by product comparison

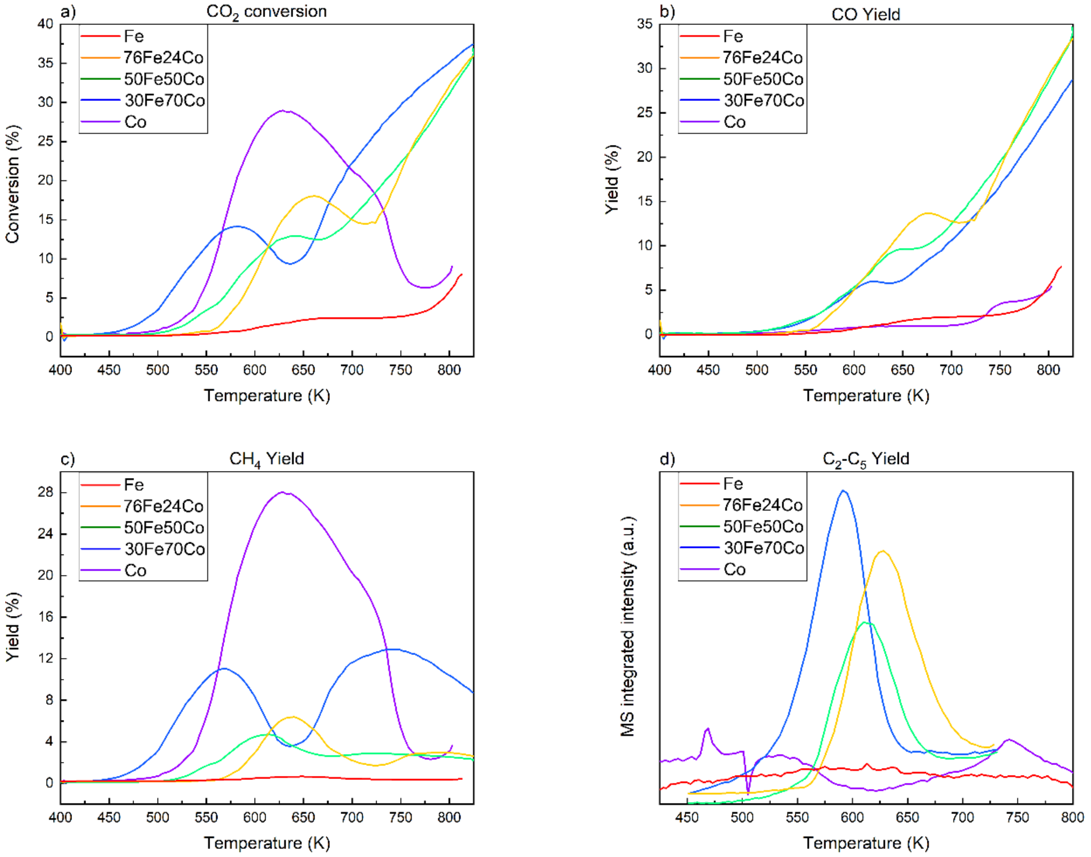

Figure S1. Catalytic properties of Fe, Co and Fe-Co NPs in a flow reactor with 4:1 H_2:CO_2 ratio, 1 bar and 10 ml_n min^{-1}, measured by mass spectroscopy. (**a**) CO_2 conversion (**b**) CO yield, (**c**) CH_4 yield (**d**) conversion curves of the summed up C2-C5 mass spectrometer normalized signals for m/z=26,29,30,39,56,57,70.

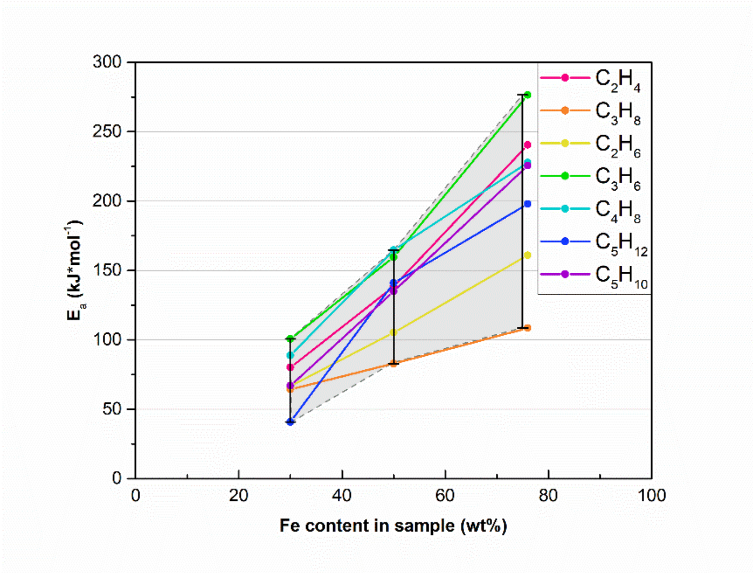

Figure S2. Activation energies of C_2-C_5 products formation as a function of the Fe content in the alloy precursor. The colours corresponding to the different products are listed in the legend, with the m/z ratio of the MS reference peak increasing from top to bottom.

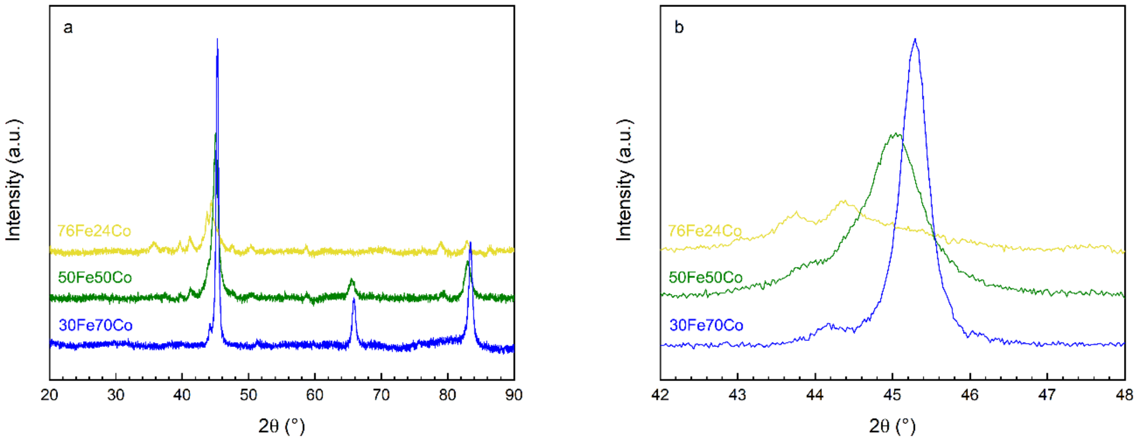

Figure S3. (**a**) XRD of the Fe-Co samples after the CO_2 hydrogenation experiments, background corrected; (**b**) a detail of the main FeCo *bcc* peak shifted to higher angular positions with increasing Co content.
